# NEUROPSYCHOLOGICAL ASSESSMENT OF POSTERIOR CORTICAL ATROPHY: A CASE STUDY

**DOI:** 10.1093/geroni/igz038.3177

**Published:** 2019-11-08

**Authors:** Amy Albright, John Burkhardt, Catherine Ikard, Anne Halli-Tierney

**Affiliations:** 1 University of Alabama, Tuscaloosa, Alabama, United States; 2 The University of Alabama College of Community Health Sciences, Tuscaloosa, Alabama, United States

## Abstract

The following case study examines the presentation of Mr. Fraser*, an older adult African American male diagnosed with Posterior Cortical Atrophy (PCA) following neuropsychological evaluation. PCA is a rare variant of Alzheimer’s Disease (AD) that results in visuospatial and perceptual deficits. Unlike other forms of neurocognitive degeneration, PCA tends to present at a relatively young age and may progress rapidly. There is currently a lack of studies examining PCA from a neuropsychological perspective, which may contribute to low awareness of this condition, as well as delayed diagnosis. It has been estimated that approximately 5% of patients with AD exhibit the PCA variant, implying that this a rare but serious condition. The following case study focuses on Mr. Fraser, a 65-year-old who was referred for neuropsychological assessment to assess his cognitive functioning. Mr. Fraser was administered a comprehensive assessment battery, and his overall results were suggestive of severe deficits in delayed memory and visuospatial skills. In the case of Mr. Fraser, these observed deficits, along with identification of visual complaints noted by his geriatrician, ultimately led to a diagnosis of PCA. While this was supported by neurological testing, the DSM-5 does not currently recognize PCA as a diagnosis. As a result, Mr. Fraser was given a diagnosis of possible AD with potential PCA, which may contribute to underestimates of the prevalence of this disorder. Future research and practice should focus on common neuropsychological presentations of this condition. *Identifying information changed in accordance with HIPAA guidelines

